# The effect of physical activity intervention and detraining on postmenopausal osteopenia and osteoporosis: a systematic review

**DOI:** 10.3389/fspor.2025.1655404

**Published:** 2025-09-22

**Authors:** Tatiana Gombarčíková, Lenka Svobodová, Aneta Svobodová, Marta Gimunová

**Affiliations:** Department of Physical Activities and Health – Faculty of Sports Studies, Masaryk University, Brno, Czechia

**Keywords:** postmenopausal women, bone health, physical activity, osteoporosis prevention, bone mineral density, training effects, exercise interventions, detraining

## Abstract

**Introduction:**

Osteoporosis is a major health concern in postmenopausal women, and regular exercise is considered a key non-pharmacological strategy for preventing its progression. The aim of this systematic review was to evaluate the effects of physical activity and subsequent detraining on bone mineral density (BMD) in this population.

**Methods:**

This systematic review was conducted in accordance with the Preferred Reporting Items for Systematic Reviews and Meta-Analyses (PRISMA) guidelines. Three databases (PubMed, Web of Science, and Cochrane Library) were searched. A total of 1,161 studies were identified, of which 3 met the inclusion criteria.

**Results:**

The findings suggest that regular resistance, aerobic, and HIIT training (2–5 sessions per week) can significantly improve or maintain BMD, particularly in the lumbar spine and proximal femur. Conversely, the cessation of exercise leads to a gradual decline in the achieved benefits within a few months.

**Conclusion:**

The results emphasize the importance of long-term physical activity as part of osteoporosis prevention while also highlighting the risks associated with the interruption of structured training.

## 1 Introduction

Osteoporosis is a systemic metabolic skeletal disease characterized by reduced bone mass and disruption of bone microarchitecture, leading to increased bone fragility and a higher risk of fractures. Osteopenia is the precursor to osteoporosis (1).

Osteoporosis has three stages: (i) osteopenia, characterized by lower bone density; (ii) osteoporosis, and (iii) severe or advanced osteoporosis. Osteopenia represents a mild loss of bone mass, whereas osteoporosis is characterized by a significant loss of bone mass and a high risk of fractures (2). Osteoporosis is further divided into primary and secondary osteoporosis. Primary osteoporosis is divided into two types (Type I and II), and idiopathic. Type I, the postmenopausal osteoporosis, is typical for women aged 55–65 years, and is related to a decrease in the level of estrogen hormones. Type II, the senile osteoporosis, it is typical for patients over 70 years of age, while women are affected twice as often as men. Idiopathic osteoporosis can occur in any age group, and its cause remains unknown (3). Secondary osteoporosis arises due to an underlying disease, most often of an endocrine nature (4, 5).

Bone density is measured by densitometry, which provides T-score values. The T-score is the difference between the patient's bone density and the reference value (norm), which is 0. When the T-score ranges from −1.0 to −2.5, it is osteopenia. A T-score below −2.5 means osteoporosis (2). Epidemiological data show that up to one in three women over the age of 50 will suffer an osteoporotic fracture during their lifetime, which significantly affects their quality of life and mortality (6). The non-pharmacological interventions of osteoporosis consist of multidisciplinary programs including vitamin D, calcium and/or protein supplementation, supervised group exercise programs, proprioceptive/vestibular retraining exercises, physical therapy (gait and balance training), hip protection, and occupational therapy (7, 8). As the key non-pharmacological tool in preventing and treating osteoporosis is considered physical activity (9).

Following classification of physical activity is often used in studies focused on osteoporosis prevention and treatment: (1) aerobic/endurance exercise—continuous, rhythmical activities that mainly stress the cardiovascular system (e.g., brisk walking, cycling); (2) weight-bearing and impact exercise—activities where the body moves against gravity and produces ground-reaction forces that load the skeleton (e.g., stair climbing, jogging, hopping/jumping); and (3) resistance/strength training—planned loading of muscles using external or body-weight resistance, usually prescribed in sets and repetitions relative to an individual's strength (10–14). The weight-bearing and impact exercise, resistance/strength training and their combination were reported to have a positive effect on bone density in postmenopausal women with no significant differences between the different types of exercise (15). Mechanical loading of bones through resistance training, impact exercises, or coordinated movement programs stimulates osteoblastic activity and supports the maintenance or increase of bone density (10–14). In addition to its direct impact on skeletal structure, regular exercise also improves muscle strength, balance, and proprioception, which reduces the risk of falls, a significant cause of osteoporotic fractures (16). Balance and flexibility exercises are considered supportive methods that primarily reduce fall risk and maintain joint range of motion rather than directly increasing bone mineral density (9, 17).

While the effects of regular physical activity on bone health are well documented, relatively little attention has been paid to the issue of detraining—periods of reduced or completely discontinued physical activity. Available evidence suggests that positive adaptations of bone tissue to exercise are time-limited and may be gradually lost with prolonged detraining (18, 19). This phenomenon is particularly important in postmenopausal women, whose bone metabolism naturally shifts toward increased resorption due to estrogen deficiency (20).

This systematic review aims to analyze and synthesize the available scientific knowledge on the effects of physical activity and subsequent detraining on bone mineral density (BMD) in postmenopausal women with osteoporosis and osteopenia. Attention will be paid to the types and intensity of exercise, the frequency of training, and the duration of intervention and detraining. The findings of this work may contribute to more effective recommendations in the secondary prevention of osteoporosis and osteopenia, as well as the optimization of long-term exercise interventions for this at-risk population.

## 2 Materials and methods

This systematic review was designed and conducted according to the Preferred Reporting Items for Systematic reviews and Meta-Analyses 2020 (PRISMA) (21). This study aimed to search and analyze studies that investigated the effects of an intervention combining physical activity and detraining on osteopenia and osteoporosis in postmenopausal women.

### 2.1 Search strategy

The acronym PICOS were used in the strategy search corresponds to P—Population: Older women with postmenopausal osteopenia and osteoporosis; I—Intervention: Physical activity intervention combined with detraining period; C—Comparison: differencies between exercise group (EG) and control group (CG); differences between the baseline, post-training, and post-detraining measures; and O—Outcome: increase, decrease or maintenance in BMD, muscle mass and function.

The bibliographic search was conducted on the following electronic databases: PubMed/ MEDLINE (National Library of Medicine), Web of Science, and Cochrane Library, including articles published up to October 2024. The keywords and boolean operators were: [(“older women” OR “elderly” OR “postmenopausal women”) AND (“osteopenia” OR “osteoporosis” OR “bone mineral density” OR “BMD” OR “areal bone mineral density” OR “bone strength” OR “bone health”) AND (“exercise” OR “strength exercise” OR “physical activity” OR “resistance exercise” OR “exercise training” OR “high-intensity resistance training” OR “HIIT” OR “aerobic training” OR “intervention” OR “exercise trials” OR “impact training” OR “low-intensity” OR “steady state training” OR “LISS” OR “exercise program” OR “exercise regime” OR “physical functional performance” OR “resistance training” OR “moderate-intensity training”) AND (“detraining” OR “training cessation” OR “exercise cessation” OR “exercise detraining” OR “inactivity” OR “training interrupted” OR “training interruption”)]. Two investigators (LS and TG) independently performed the search terms in each database, and the search agreement was verified.

### 2.2 Eligibility criteria

Studies were included according to the following criteria: (1) original articles published up to October 2024; (2) sample including older women with postmenopausal osteopenia and osteoporosis; (3) protocols including physical intervention (4) protocols including detraining period or training cessation as exposure; (5) measurement of bone parameters (e.g., x-rays, DXA, blood analysis). The experimental group and control group should receive the same testing period. Review, meta-analysis, case reports, abstracts, book chapters, opinion/position papers, editorials, and studies with no full text available and other languages than English were excluded.

### 2.3 Screening and selection

**Figure 1:**
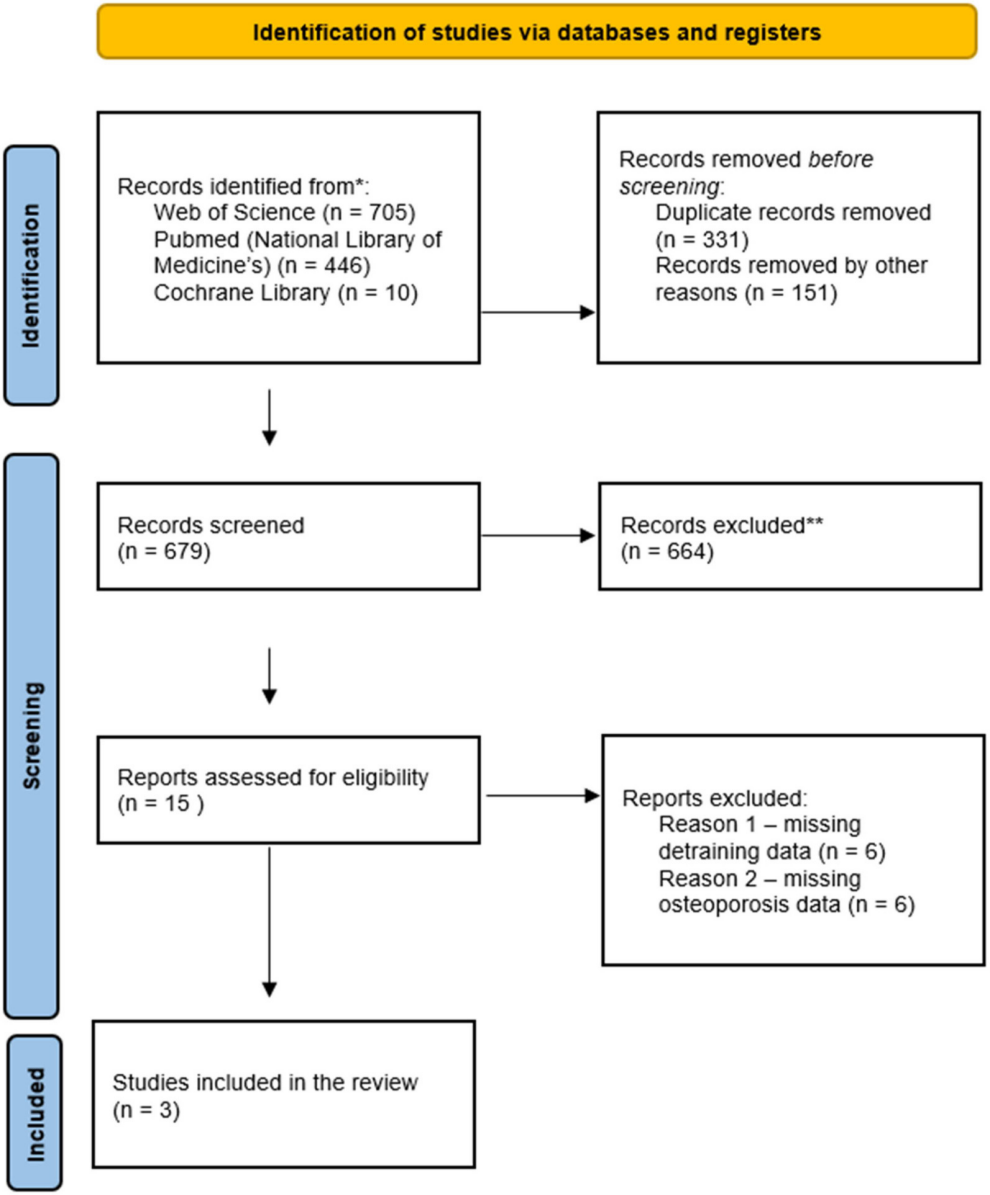
Flow chart of the systematic review according to the PRISMA statement. PRISMA, preferred reporting items for systematic reviews and meta-analyses.

The process of selecting studies was independently conducted by two investigators (LS and TG). Initially, two investigators (LS and TG) analyzed the titles and abstracts of all records. When the study met the selection criteria, the full text was analyzed. When there was a discrepancy between the two evaluators, a meeting was scheduled to decide whether to include or exclude the study. In disagreement, a third investigator (MG) was invited to decide. To illustrate the selection steps until the Inclusion of studies, Figure 1 provides a flowchart proposed by PRISMA, with the number of studies identified in the literature (Identification), screening and removal of duplicate reports, removal by title and abstract (Screening), Full studies and removal of studies by eligibility criteria and Inclusion of full studies (Inclusion). The Rayyan online software (22) was used to screen and select the studies.

### 2.4 Data extraction process

The authors created a structural form to extract the primary data from the selected studies by two independent investigators. Using a structural form, two investigators (LS and TG) extracted the following data from selected studies: author's name, publication date, country, sample characteristics, training characteristics (e.g., training type, frequency, duration, adherence), detraining characteristics (e.g., duration), BMD measurements, pre-to-post intervention results. The results are described as mean and standard deviation, when available.

### 2.5 Quality and risk of bias assessment

Study quality was assessed using an adapted version of the Downs & Black (23) based on a previous review (24), selecting relevant questions according to the study methodology. The Downs and Black checklist (23) is divided and reported into five different subscales, which comprise 27 questions on: (1) Reporting—which assessed whether the information provided in the paper was sufficient to allow a reader to make an unbiased assessment of the findings of the study. (2) External validity—which addressed the extent to which the findings from the study could be generalized to the population from which the study subjects were derived. (3) Bias—which addressed biases in measuring the intervention and the outcome. (4) Confounding addresses bias in the selection of study subjects. (5) Power, which attempted to assess whether the negative findings from a study could be due to chance. For each item, a binary score was used, in which 0 represents no/unable to determine and 1 = yes, indicating that the study presents the item. The final score of 18 selected questions was converted to percentages. The methodological quality was classified as follows: <45.4% “poor” methodological quality; 45.4%–61.0%, “fair” methodological quality; and >61.0%, “good” methodological quality (24).

## 3 Results

### 3.1 Study selection

A total of 1,161 studies were retrieved from three databases [PubMed/MEDLINE (National Library of Medicine), Web of Science, and Cochrane Library]. Of these, 331 duplicates were removed. Before screening, 151 records were excluded due to ineligible publication language (non-English). The remaining 679 records underwent title and abstract screening; 664 were excluded due to ineligible study type (reviews, meta-analyses, case reports, abstracts, book chapters, opinion/position papers, editorials), not matching the topic, or lack of full text. Fifteen articles were assessed in full. Twelve were excluded (six did not include a detraining period and six did not report osteoporosis data). Thus, this systematic review comprised three studies.

### 3.2 Study characteristics

This systematic review evaluates the effects of physical activity and detraining on osteoporosis and osteopenia in postmenopausal women. A total of three studies were included in the final review. Table 1 shows the basic characteristics of the individual studies; the number of participants ranged from 34 (25) to 54 participants (26). The age of the participants ranged from 48 (26) to 88 years (25). Two studies reported that no estrogen medication was used during the intervention (26, 27). Estrogen medication was not reported in the study by Englund et al. (25).

**Table 1:** Sample characteristics.

Study	Age (years)	Sample size (n)	Height (cm)	Weight (kg)	BMI	Time since menopause (years)	Medication of estrogen
Englund et al., (25)	73–88	EG: 18	EG: 161, 0 ± 6, 0	EG: 69, 0 ± 7, 1	EG: 25, 2 ± 2, 8	N.A.	N.A.
		CG: 16	CG: 163, 0 ± 4, 9	CG: 67, 5 ± 8, 3	CG: 26, 4 ± 2, 8		
Iwamoto et al., (27)	53–77	EG:15	EG: 152 ± 7, 84	EG: 45, 5 ± 6, 5	EG: 19, 7 ± 1, 3	EG: 16.3 ± 5.9	NO
		CG: 20	CG: 152 ± 5, 66	CG: 45, 8 ± 4, 0	CG: 19, 9 ± 2, 1	CG: 14.7 ± 9.2	
Kemmler et al., (26)	48–60	EG: 27	EG: 164, 2 ± 6, 0	EG: 64, 0 ± 9, 6	N.A.	N.A.	NO
		CG: 27	CG: 164, 5 ± 8, 2	CG: 67, 4 ± 14, 6			

Table 2 shows a description of the physical activity intervention and detraining. The physical activity intervention ranged from 12 months (25, 27) to 13 months (26). In the included studies, different training modalities were used: resistance training (25, 26), balance training (25), aerobic (25), HIIT (26), and resistance training (10 min per day) combined with daily step count (27). The training frequency ranged from 2 (25) to 5 per week (27). The detraining periods range from 3 months (26) to 5 years (25).

**Table 2:** Description of physical activity intervention and detraining.

Study	Type of intervention	Duration of intervention	Frequency weekly	Adherence	Duration of detraining
Englund et al., (25)	resistance training + aerobic + balance in 1 session	12 months	2x	N.A.	5 years
Iwamoto et al., (27)	resistance training (20 min) + step count	12 months	5x	N.A.	12 months
Kemmler et al., (26)	HIIT + high-effort resistance training	13 months	3x	79 ± 12% EG/78 ± 14% CG	3 months

Table 3 provides us with the methods used in the included studies. Blood sample analysis used two studies to determine the level of bone parameters in pre- and post-tests (25, 27). Furthermore, two studies mentioned using dietary supplements throughout the intervention period among participants (26, 27). Englund et al. (25) assessed functional performance using the maximal walking speed test over a 30-meter walkway. Standing balance was evaluated using the one-leg stance test, in which participants performed two 120-second trials with a 1-minute rest interval; the better result was used for analysis (46).

**Table 3:** Tests used to detect BMD and physical tests.

Study	BMD	BMD device	Daily supplements	Blood sample analysis	Strength test	Strength-test device(s)	Functional performance/Physical activity	Balance test
Englund et al., (25)	TB-BMC, TB-BMD (Arms, Lumbar spine, Femoral neck, Trochanter, Ward's triangle)	DXA, Lunar DPX-L (Lunar Co., Wisconsin, USA)	N.A.	25-OH Vitamin D2, 25-OH Vitamin D3, β-CTx, Osteocalcin	Hand-grip strength; Knee-extension strength (isometric)	Hand dynamometer/tensiometer (Gossen, Sweden; No. 12,016)	Maximal walking speed test (30 m corridor, “as fast as possible”)	Berg one-leg stance test
Iwamoto et al., (27)	Lumbar spine	DXA, Norland XR-26 (Fort Atkinson, Wisconsin, USA)	2 g calcium lactate + 1 μg 1α-hydroxyvitamin D3	Alkaline phosphatase, Phosphorus, Calcium	N.A.	N.A.	Weekly step count (habitual physical activity monitoring)	N.A.
Kemmler et al., (26)	Lumbar spine	DXA, QDR 4,500a, Discovery-upgrade (Hologic Inc., Bedford, USA)	800 IU vitamin D + 1,000 mg calcium	N.A.	Maximal isokinetic leg/hip-extensor strength; Lower-limb power (CMJ)	Isokinetic leg press CON-TREX LP (Physiomed, Laipersdorf, Germany); Force platform (KMP Newton GmbH, Stein, Germany)	N.A.	N.A.

Muscle strength assessments were specified as follows. Isometric handgrip strength was assessed only in Englund et al. (25) using a hand-held dynamometer (Gossen, Sweden; No. 12016). Lower-limb strength was evaluated in Englund et al. (25) and Kemmler et al. (26), but with different protocols and devices: Englund et al. (25) measured isometric knee extensor strength with the dynamometer (Gossen, Sweden; No. 12016), whereas Kemmler et al. (26) assessed maximal isokinetic leg/hip extensor strength using an isokinetic leg press (CON-TREX LP, Physiomed, Laipersdorf, Germany). In addition, Kemmler et al. (26) evaluated lower-limb power via a countermovement jump (CMJ) performed on a force platform (KMP Newton GmbH, Stein, Germany).

Table 4 shows results of the included studies describing training outcomes (difference between baseline measure and post-training intervention), detraining outcomes (difference between post-training intervention and post-detraining intervention), and baseline outcomes (difference between baseline and post-detraining intervention).

**Table 4:** Table showing the outcomes of the studies.

Study	Training outcome			Detraining outcome			Difference from baseline		
	Strength after intervention	Endurance after intervention	BMD after intervention	Strength after detraining	Endurance after detraining	BMD after detraining	Strength	Endurance	BMD
Englund et al., (25)	↑EG	↑EG	↑EG	↓EG	↓EG	∼EG (LS)	∼EG	∼EG	↓EG
	↓CG	↓CG	∼CG	↓CG	↓CG	↑CG (LS)	∼CG	∼CG	∼CG
Iwamoto et al., (27)	N.A.	↑EG	↑EG	N.A.	↑EG	↑EG	N.A.	∼EG	N.A.
		∼CG	∼CG		∼CG	∼CG		∼CG	
Kemmler et al., (26)	↑EG	N.A.	∼EG	↓EG	N.A.	∼EG	∼EG	N.A.	∼EG
	↑CG		↓CG	↓CG		∼CG	∼CG		∼CG

EG, exercise group; CG, control group; LS, lumbar spin.

All three studies adjusted for the intervention effect and showed that the training group improved in strength and functional tests compared to the control group. In the control group, functional performance, strength, or power either did not change significantly from the initial testing or even deteriorated below the initial testing value (25–27).

The exercise intervention also had a positive effect on BMD. Englund et al. (25) and Iwamoto et al. (27) confirmed a statistically significant improvement in bone density after the intervention, but no change in the control group. On the other hand, Kemmler et al. (26) did not achieve significant results in bone density in the exercise group, and in the control group, there was a deterioration.

Englund et al. (25), Kemmler et al. (26), and Iwamoto et al. (27) tested strength and functional performance. Englund et al. (25) and Kemmler et al. (26) observed the predicted effect of detraining; functional performance and/or strength in their studies were worse after the end of detraining compared to the end of the intervention. In the two studies mentioned above, we can observe deterioration in the control groups. In Iwamoto et al. (27), there was an improvement in the monitored functional indicator (weekly step count) after the end of the detraining phase in the experimental group, and no change in the control group.

There was no effect of detraining on BMD in the exercise group; only 1 study showed an improvement after detraining (27). In the control groups, there was no improvement in BMD after detraining in Iwamoto et al. (27) and Kemmler et al. (26), whereas Englund et al. (25) showed an improvement.

No significant functional or strength or BMD changes were observed in any group comparing post-detraining and baseline testing in Iwamoto et al. (27) and Kemmler et al. (26). On the other hand, Englund et al. (25) noted a deterioration in BMD after detraining compared to baseline in the exercise group, while the control group improved (25).

## 4 Discussion

This systematic review analyzed the impact of exercise and subsequent detraining on bone health in postmenopausal women with osteopenia and osteoporosis. Despite the high number of research studies focused on physical activity in older adults and osteoporosis, studies specifically addressing the effects of detraining remain scarce. Results from the three identified studies demonstrated significant benefits of physical activity on BMD (25, 27), strength (25, 26), and functional performance/habitual activity (25, 27). However, the results concerning the detraining period indicated that the positive effects of exercise on BMD are not permanent and may be time-limited. Strength and functional performance generally declined after detraining, although results related to BMD varied. Due to the small number of included studies in this systematic review, the generalization of their findings is limited.

Englund et al. (25) observed that a 12-month exercise intervention significantly improved BMD, yet after five years without structured exercise, these improvements disappeared entirely. Conversely, Iwamoto et al. (27) found stable or slightly improved BMD following a 12-month detraining period, indicating individual or methodological factors variability. Kemmler et al. (26) noted significant enhancements in strength and power without significant changes in BMD during 13 months of training. However, after three months of detraining, significant muscle mass and performance decreases occurred while BMD remained unchanged.

The exercise protocols differed in several key aspects that likely contribute to the heterogeneous findings. In the study by Englund et al. (25), participants completed a 12-month supervised, combined weight-bearing program (resistance, aerobic, and balance/coordination) twice weekly for 50 min, with ∼67% adherence among participants, with the BMD gains dissipating after ≈5 years without structured training. In the study by Kemmler et al. (26), early-postmenopausal osteopenic women undertook a 13-month supervised, multipurpose high-impact weight-bearing + high-intensity/velocity resistance program with HIIT and a jump sequence, performed 3×/week (≈40 min twice in lab, and ≈60 min once in a gym), with adherence ∼79% and standardized cholecalciferol and calcium supplementation resulting in BMD unchanged in the exercise group (and decreased in controls). The study by Iwamoto et al. (27) incorporated daily calcium and active vitamin D (1α-OH-D3) supplementation and monitored habitual physical activity (weekly step count), reporting a statistically significant improvement in lumbar-spine BMD after training and stability/slight improvement after 12 months of detraining.

The stable or slightly improved BMD after detraining in Iwamoto et al. (27) likely reflects co-interventions and measurement factors: all participants—including during the 12-month detraining phase—received daily calcium and 1α-hydroxyvitamin D3 supplementation, which can help maintain BMD. Additionally, the lumbar-spine DXA site has a relatively small least significant change (LSC) (28), so small positive shifts may be detectable. Additionally, delayed/secondary mineralization can sustain BMD for some time after the structured training stops. The authors did not report pedometer-based step counts or quantified activity during detraining, so any maintenance of habitual activity is inferred rather than documented. Taken together, these points plausibly account for stable or slightly higher lumbar BMD after 12 months of detraining (27–30).

Adherence is a key determinant of bone outcomes. In Dalsky et al. (31), >90% attendance yielded up to a 6.1% lumbar BMD gain over 22 months, with partial regression after 13 months of detraining; by contrast, Kemmler et al. (26) reported ∼78%–79% attendance and no significant BMD change despite strength/power gains. Consistent with meta-analyses, programs with adequate “dose” (progressive resistance, ≥2 sessions/week, sustained ≥9–12 months) and high compliance produce larger BMD effects; trials should target ≥80%–90% adherence (12, 14).

A study by Havill et al. (32) examined the effects of genes, sex, age, and physical activity on BMD. After accounting for the effects of age, sex, and activity level, genes explained 40%–62% of the residual variation in BMC and BMD and 27%–75% in bone size. With age, the decline in areal BMD of the femoral neck, hip, and spine was greater in women than in men. Younger women had higher cortical volumetric BMD than younger men, with minimal difference between low and high activity levels. The effect of activity was greater in older women: older women with low activity had lower cortical volumetric BMD than older men, but older women with high activity had higher cortical volumetric BMD than older men.

Another important aspect is the age variability among study populations. Age differences of participants (from 48–88 years) across the included studies might influence the responsiveness of bone tissue to mechanical loading and the rate of subsequent adaptation decline during detraining. This underscores the need for tailored exercise protocols adjusted to the participants' age and health status. Studies by Meyer et al. (33) showed that regular physical activity can positively influence BMD in prepubertal and early pubertal boys and girls. A multi-component school-based physical activity intervention lasting one academic year simultaneously improved bone health in primary school children.

Comparable studies on combined aerobic and resistance training (RT) highlight varied results regarding detraining effects. For example, Douda et al. (34) implemented four cycles of 9-month training followed by 3-month detraining periods, noting regular non-significant decreases in lean body mass (LBM), returning consistently to pre-training levels in women over 60. Only in one 9-month cycle was there a noticeable increase in LBM. In contrast, Bickel et al. (35), who conducted a shorter (16-week) intensive RT focused on lower limbs in older adults (60–75 years), observed significant reductions in thigh lean mass after just 8 weeks of detraining.

However, these findings are not conclusive. Lovell et al. (36) and Fiatarone et al. (37) documented significant reductions in maximal leg strength (1 RM squat) after only 4 weeks of detraining in older populations. However, these remained higher compared to baseline levels. Conversely, Hakkinen et al. (38), in a 24-week RT program involving middle-aged (37–44 years) and older adults (63–78 years), found no significant declines in neuromuscular performance after a shorter, 3-week detraining period, suggesting the stability of adaptations might be related to training duration as well as detraining length.

Research on detraining is scarce, mainly due to the need for more extended training periods, given that bone metabolism is slower and training effects on bones are less pronounced than muscle mass or strength (30, 39). Similar studies applying long training and detraining periods in young women and elite female gymnasts confirmed our findings of mild but non-significant decreases in BMD after detraining (40, 41). Other studies indicate significant BMD and muscle mass decreases after detraining (26). Taken together, protocol content (resistance vs. impact components), duration and intensity, adherence, nutritional co-interventions, and measurement site collectively shape whether BMD is maintained, improves modestly, or declines during and after detraining. The optimal detraining duration that allows regeneration without adverse effects remains unclear. Some studies suggest short-term interruptions (5–6 weeks) for regeneration, maintaining at least one-third of the original training volume to preserve acquired adaptations (35, 42). Strength exercise is a powerful stimulus to improve and maintain bone mass during the aging process. Multi-component exercise programs of strength, aerobic, high-impact, and/or weight-bearing training, as well as whole-body vibration alone or in combination with exercise, may help to increase or at least prevent decline in bone mass with aging, especially in postmenopausal women (43). Unsupervised exercise has also improved bone and lower limb BMD in adult women. The beneficial effects of exercise on femoral neck and lumbar spine BMD may be more pronounced in women with poor bone health than in healthy women (44).

Based on findings, it is recommended to continue regular, long-term physical activity as part of secondary osteoporosis prevention, emphasizing the minimization of detraining periods. Practical strategies to support continuous exercise among older women are crucial to maintaining long-term health benefits. Effective delivery should involve specialised exercise professionals (e.g., physiotherapists/clinical exercise physiologists) to individualise progressive resistance and impact loading, coach safe spine mechanics, and monitor adherence (10, 16). It is also important to ensure adequate calcium and vitamin D supplementation with the treating physician. To limit detraining losses, promoting habitual daily living activity (e.g., step counts) is recommended, as higher steps/day are associated with healthier bone in older women. When breaks in training are unavoidable, including maintenance phases preserving roughly one-third of the training dose to retain adaptations is recommended (35, 45).

## 5 Limitations

A key limitation of this study was the limited number of relevant studies identified—only three, despite the absence of any restriction on the year of publication. The literature search was conducted using three major scientific databases. Although extensive research exists on physical activity in older adults and osteoporosis, studies specifically addressing the effects of detraining remain scarce, as evidenced by the findings of this systematic review. Furthermore, considerable variability in the type and duration of both the interventions and the detraining periods may have introduced inconsistencies, potentially affecting the validity and comparability of the results.

Limited knowledge exists on strategies to minimize detraining effects and sustain adherence to physical activity among older women. Further research should clarify optimal detraining duration, the combined effects of detraining with calcium and vitamin D supplementation, and the role of habitual daily activity (e.g., step counts) in maintaining skeletal adaptations during detraining.

## 6 Conclusion

In conclusion, regular exercise significantly improves BMD, muscular strength, and endurance in postmenopausal women with osteopenia and osteoporosis. However, these improvements are at risk during detraining, suggesting continuous physical activity is essential to maintain these benefits. Further research is needed to determine optimal exercise prescriptions and strategies for effectively managing periods of reduced physical activity. Tailored exercise interventions focusing on adherence, intensity, duration, and specific individual needs are recommended to sustain long-term bone health and physical function in older women.

## Data Availability

The original contributions presented in the study are included in the article/Supplementary Material, further inquiries can be directed to the corresponding author.
